# Supplementary material for: Investigation of Molecular Weight, Polymer Concentration and Process Parameters Factors on the Sustained Release of the Anti-Multiple-Sclerosis Agent Teriflunomide from Poly(ε-caprolactone) Electrospun Nanofibrous Matrices
Source: Pharmaceutics. 2022 Aug 14;14(8):1693. doi: 10.3390/pharmaceutics14081693 (PMC9412398; doi:10.3390/pharmaceutics14081693)
Supplement: Supplementary file 1 [file pharmaceutics-14-01693-s001.zip › pharmaceutics-1848156-supplementary.pdf]

Supplementary Material

For

Investigation of molecular weight, polymer concentration and process parameters factors on the sustained release of the anti-multiple sclerosis agent teriflunomide from poly(ε-caprolactone) electrospun nanofibrous matrices

S1.Nuclear magnetic spectroscopy (NMR) characterization

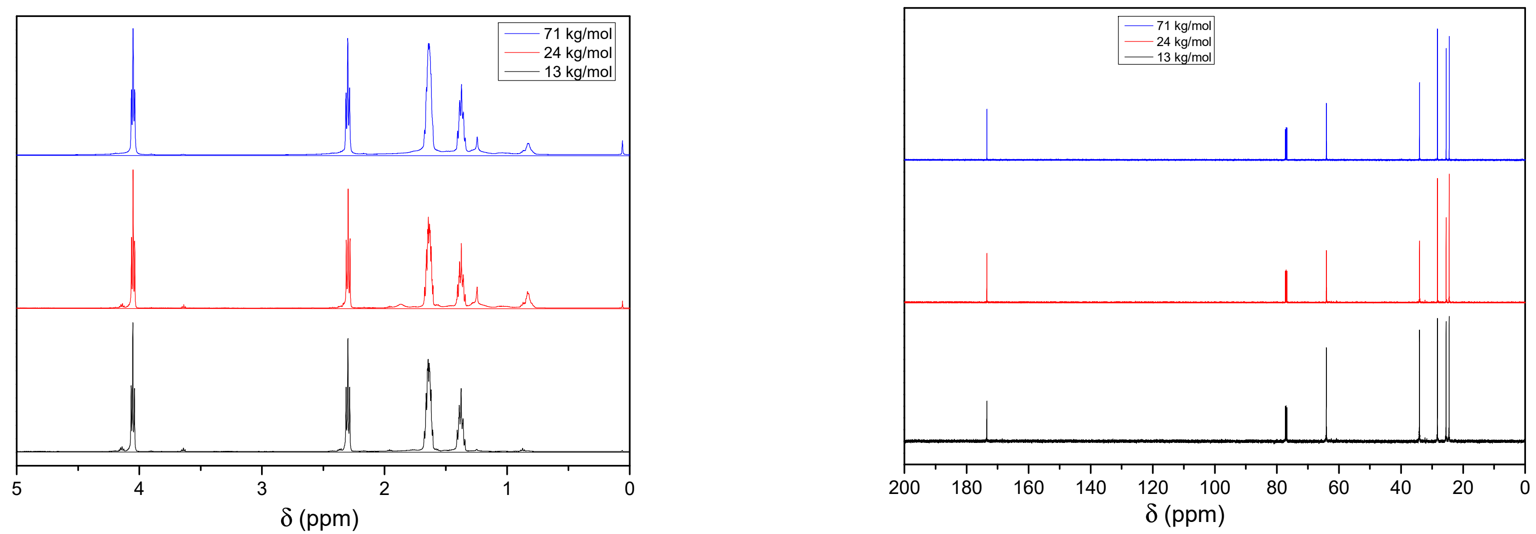

Figure S1. ^{1}H (left) and ^{13}C (right) spectra of the synthesized PCLs. Solvent residual peaks: 7.26 (^{1}H) and 77.0 (^{13}C) ppm.

NMR spectra are typical for PCL. In the ^{1}H spectrum, the resonance signals at 4.06 and 2.30 ppm correspond to the methylene groups adjacent respectively to the O atom and to the C=O group of the ester moiety. The multiple peaks at 1.65 and 1.38 ppm that complete the spectrum, are attributed to the three remaining methylene groups of the structure. In the ^{13}C spectrum, the C=O carbon atom is clearly observed at 173.5 ppm. The methylene groups adjacent to the O atom and C=O group of the ester moiety are observed at 64.1 and 34.0 ppm respectively. The resonance signals at 28.3, 25.5 and 24.5 correspond to the three remaining methylene groups.

S2. Effect of initiator to the molecular weight of PCL polyesters.

SEC technique was employed here to determine the samples molar mass, Mn and Mw, as well as the corresponding dispersity(Figure 2).The synthesized polymers had average molecular weights (Mn) ranging from 13.400 to 70.976 g/mol and dispersity in the range of 1.4–1.6 using increasing monomer/initiator molar ratios.

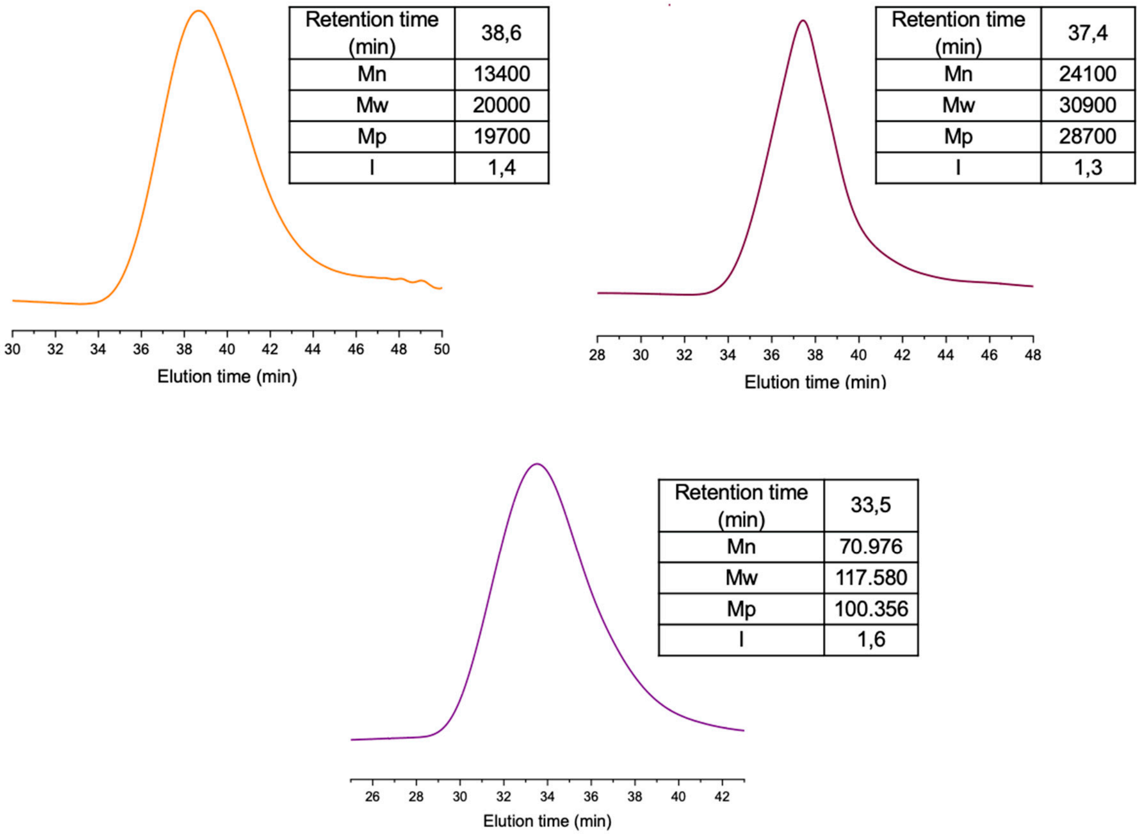

Retention time (min)	38,6
Mn	13400
Mw	20000
Mp	19700
I	1,4

Retention time (min)	37,4
Mn	24100
Mw	30900
Mp	28700
I	1,3

Retention time (min)	33,5
Mn	70.976
Mw	117.580
Mp	100.356
I	1,6

Figure S2. Raw data by size-exclusion chromatography, including the estimation of molar masses and the dispersity indices.

From a literature study of previous studies, it was concluded that by varying the type and concentration of the initiator (i.e. the number of hydroxyl in alcohol), polymers with different chain characteristics and molecular weights could be prepared. In

particular, in the presence of mono- and bi-functional alcohols, linear polymer chains were produced while in the presence of polyalcohols with more than two hydroxyl groups, a solid and branched polymer was obtained. In addition to the starting role of chain water supply, the hydroxyl groups can also act as chain transfer agents.Therefore, it is very important to accurately control the concentration of hydroxyl species as they greatly affect the polymerization rate and molecular weight of PCL. It has also been observed that as the concentrator concentration increases (i.e., the value of the [M] / [CI] ratio decreases), the polymerization rate increases. The value of Mn shows a linear increase with respect to the conversion of monomers, a phenomenon characteristic of "ideal living" polymerization, as the number of developing polymer chains remains constant until very high conversion of monomers.

In addition, from the study conducted by Karidi et al[1]it was showed that, when the ratio [M] / [ROH] is 5,000 and 10,000, the molecular weight increases with respect to the case where alcohol is not used as a initiator. In contrast, for high concentrations of alcohol ([M]/[ROH] = 2,000) the molecular weight decreases. This is attributed to the large concentrations of low molecular weight polymer chains produced at high concentrations of the initiator. In the present work the above finding is fully confirmed since the dependence of the molecular / monomer ratio of the molecule with the final molecular weight is observed. More specifically, reducing the amount of butanediol (thus increasing the monomer / alcohol molar ratio) leads to an increase in the final molecular weight of the polymer.

S3. Morphological characterization of all examined systems

Table S1. SEM images of the examined process conditions of 13.400 Mn PCL in 12% w/v in chloroform.

Sample	SEM	Sample	SEM	Sample	SEM	Sample	SEM
1	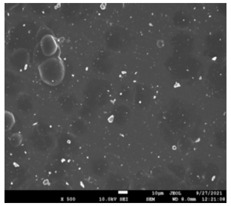	4	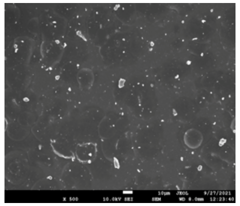	7	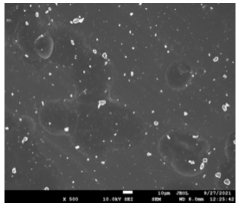	10	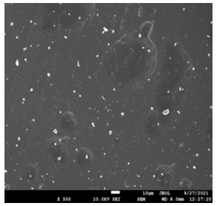
2	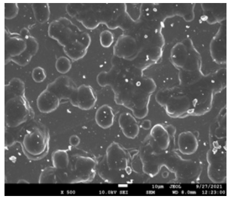	5	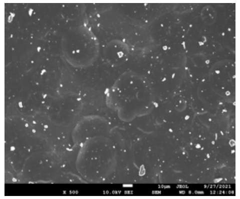	8	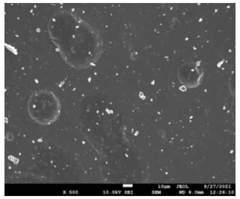	11	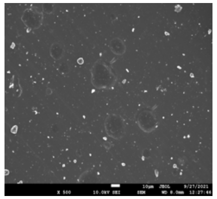
3	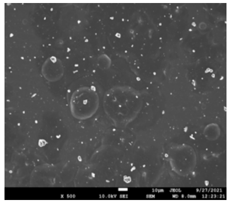	6	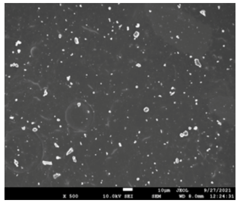	9	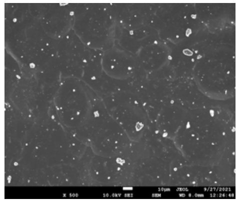	12	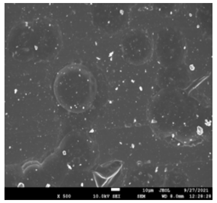

Table S2. SEM images of the examined process conditions of 13.400 Mn PCL in 15% w/v in chloroform.

Sample	SEM	Sample	SEM	Sample	SEM	Sample	SEM
1	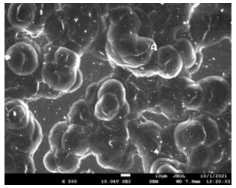	4	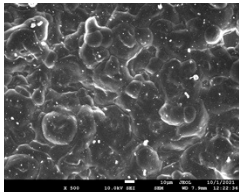	7	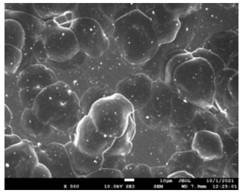	10	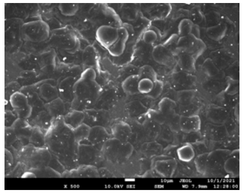
2	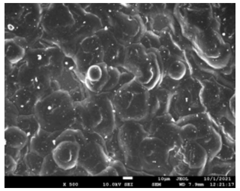	5	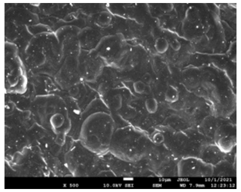	8	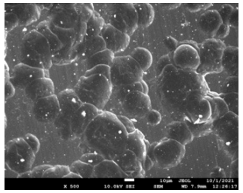	11	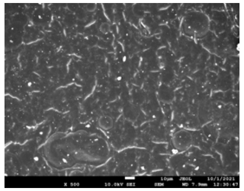
3	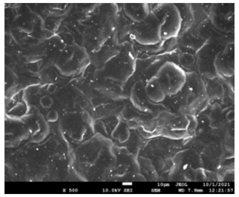	6	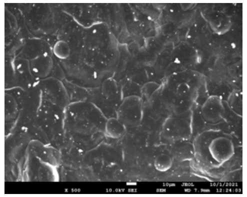	9	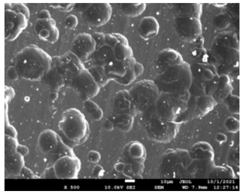	12	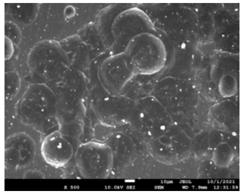

Table S3. SEM images of the examined process conditions of 13.400 Mn PCL in 17% w/v in chloroform.

Sample	SEM	Sample	SEM	Sample	SEM	Sample	SEM
1	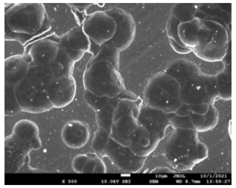	4	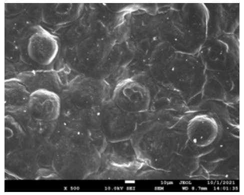	7	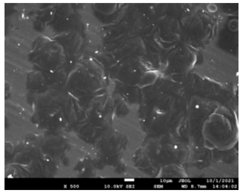	10	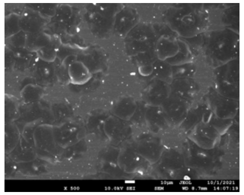
2	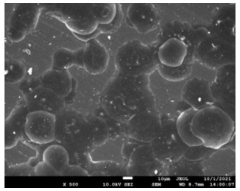	5	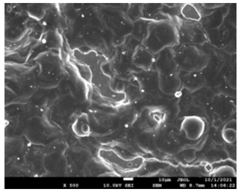	8	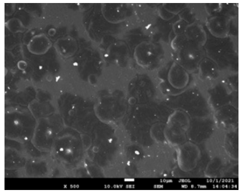	11	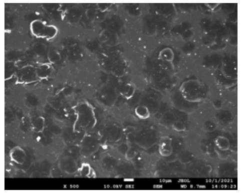
3	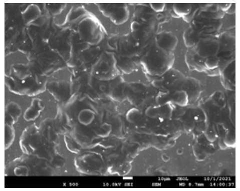	6	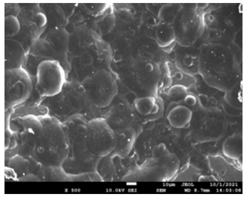	9	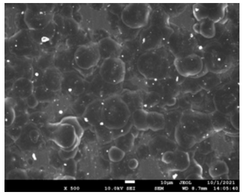	12	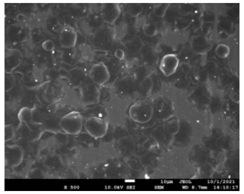

Table S4. SEM images of the examined process conditions of 24.100 Mn PCL in 12% w/v in chloroform.

Sample	SEM	Sample	SEM	Sample	SEM	Sample	SEM
1	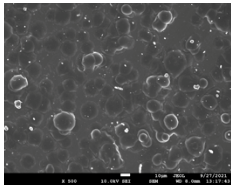	4	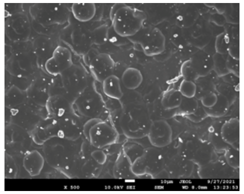	7	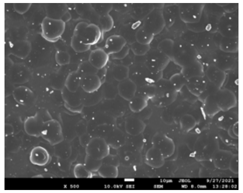	10	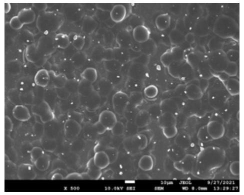
2	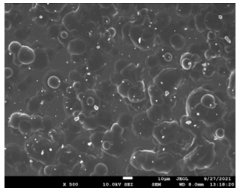	5	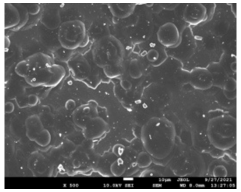	8	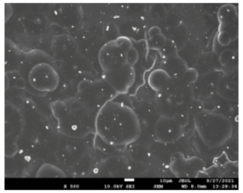	11	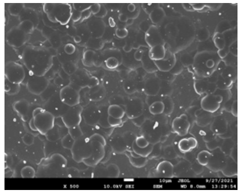
3	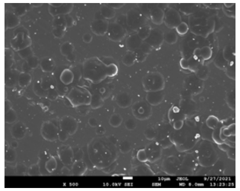	6	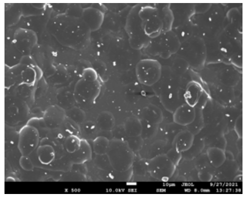	9	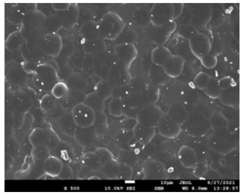	12	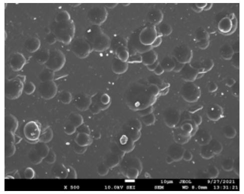

Table S5. SEM images of the examined process conditions of 24.100 Mn PCL in 15% w/v in chloroform.

Sample	SEM	Sample	SEM	Sample	SEM	Sample	SEM
1	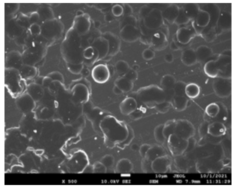	4	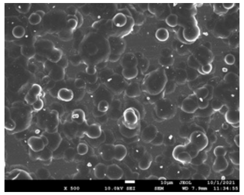	7	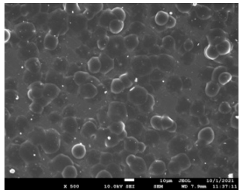	10	-
2	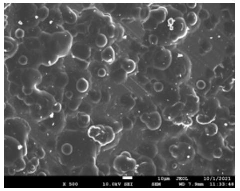	5	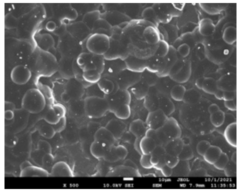	8	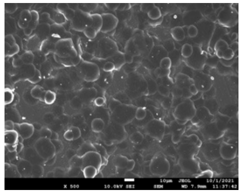	11	-
3	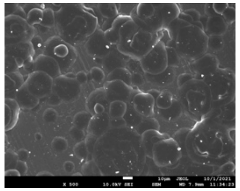	6	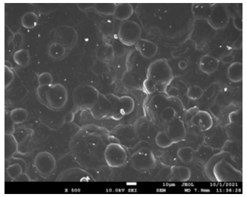	9	-	12	-

Table S6. SEM images of the examined process conditions of 24.100 Mn PCL in 17% w/v in chloroform.

Sample	SEM	Sample	SEM	Sample	SEM	Sample	SEM
1	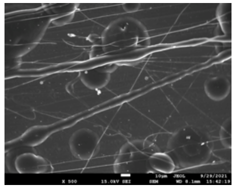	4	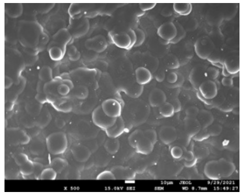	7	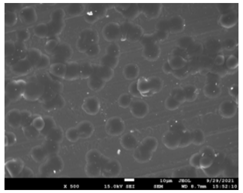	10	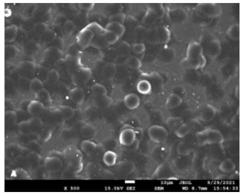
2	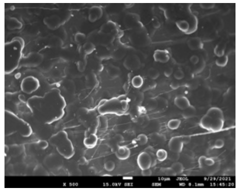	5	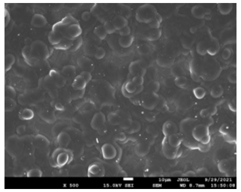	8	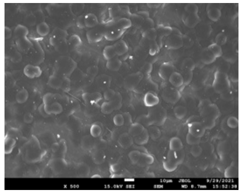	11	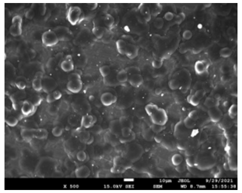
3	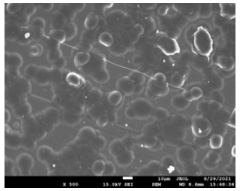	6	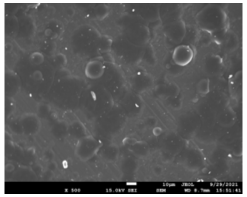	9	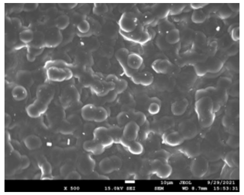	12	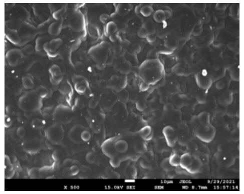

Table S7. SEM images of the examined process conditions of 70.976 Mn PCL in 12% w/v in chloroform.

Sample	SEM	Sample	SEM	Sample	SEM	Sample	SEM
1	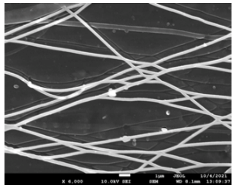	4	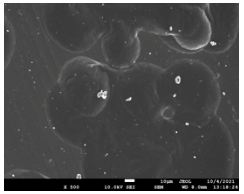	7	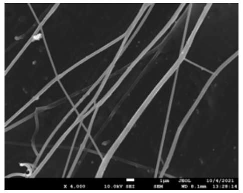	10	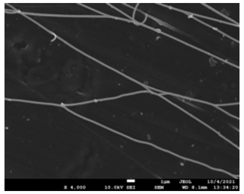
2	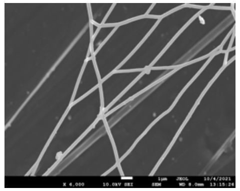	5	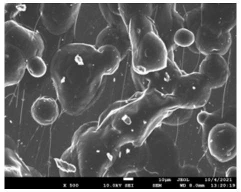	8	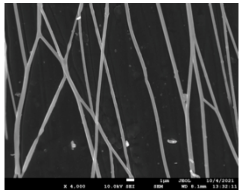	11	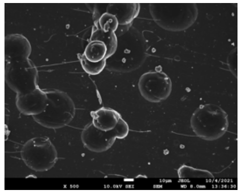
3	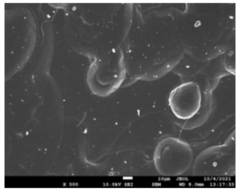	6	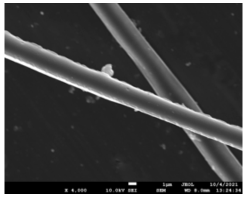	9	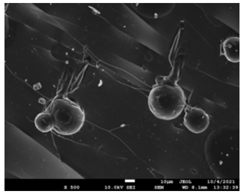	12	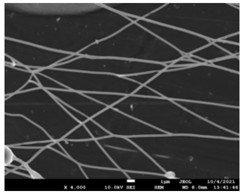

Table S8. SEM images of the examined process conditions of 70.976 Mn PCL in 15% w/v in chloroform.

Sample	SEM	Sample	SEM	Sample	SEM	Sample	SEM
1	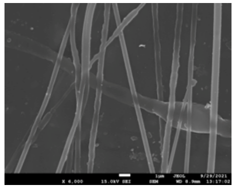	4	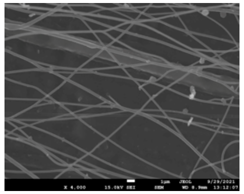	7	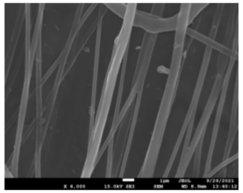	10	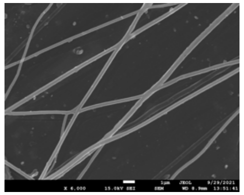
2	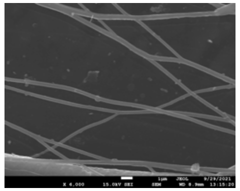	5	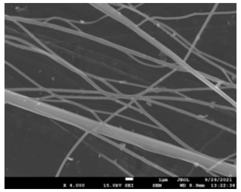	8	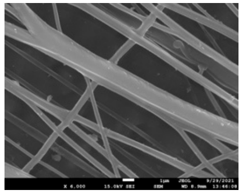	11	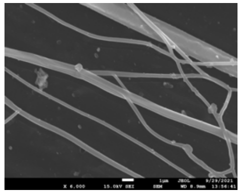
3	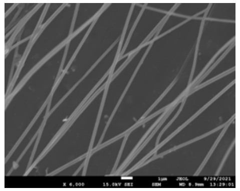	6	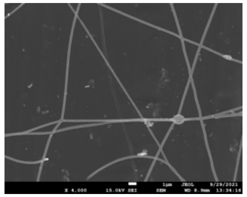	9	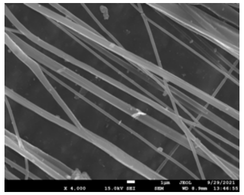	12	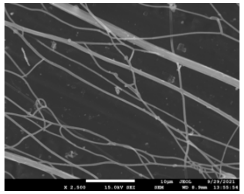

Table S9. SEM images of the examined process conditions of 70.976 Mn PCL in 17% w/v in chloroform.

Sample	SEM	Sample	SEM	Sample	SEM	Sample	SEM
1	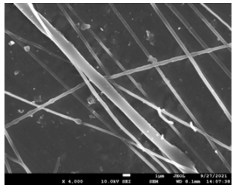	4	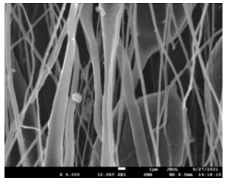	7	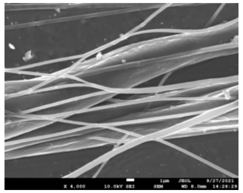	10	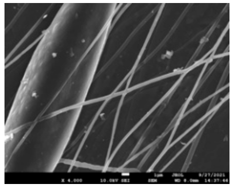
2	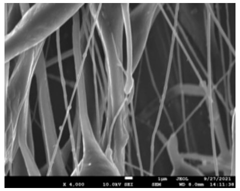	5	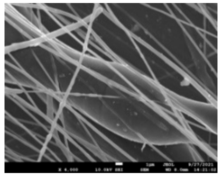	8	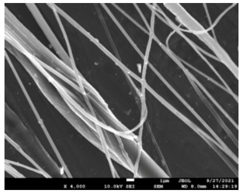	11	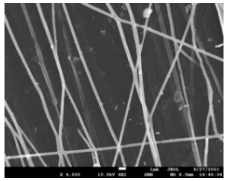
3	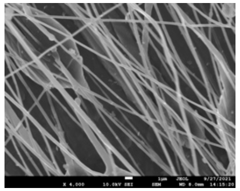	6	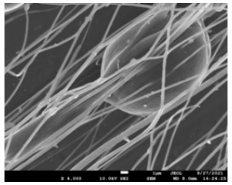	9	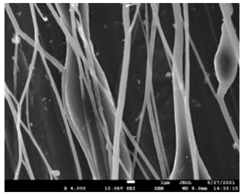	12	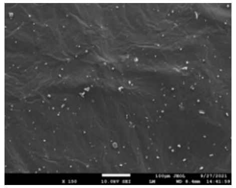

Reference

[1] Karidi, K.; Mantourlias, T.; Seretis, A.; Pladis, P.; Kiparissides, C. Synthesis of High Molecular Weight Linear and Branched Polylactides: A Comprehensive Kinetic Investigation. *Eur. Polym. J.* **2015**, *72*, 114–128. https://doi.org/10.1016/j.eurpolymj.2015.09.011.
